# Monohexosylceramides from *Rhizopus* Species Isolated from Brazilian Caatinga: Chemical Characterization and Evaluation of Their Anti-Biofilm and Antibacterial Activities

**DOI:** 10.3390/molecules23061331

**Published:** 2018-06-01

**Authors:** Edson Rodrigues Vieira, Mariana Ingrid Dutra da Silva Xisto, Milagre Américo Pele, Daniela Sales Alviano, Celuta Sales Alviano, Eliana Barreto-Bergter, Galba Maria de Campos-Takaki

**Affiliations:** 1 Núcleo de Pesquisa em Ciências Ambientais e Biotecnologia, Universidade Católica de Pernambuco, Recife 50050-590, PE, Brazil; edsonipubi@gmail.com (E.R.V.); phelema1@gmail.com (M.A.P.); galba_takaki@yahoo.com.br (G.M.d.C.-T.); 2 Laboratório de Química Biológica de Microrganismos, Instituto de Microbiologia Paulo de Góes, Centro de Ciências da Saúde, Universidade Federal do Rio de Janeiro, Ilha do Fundão, Rio de Janeiro 21941-902, RJ, Brazil; marylanax@gmail.com; 3 Laboratório de Estrutura de Microrganismos, Instituto de Microbiologia Paulo de Góes, Centro de Ciências da Saúde, Universidade Federal do Rio de Janeiro, Ilha do Fundão, Rio de Janeiro 21941-902, RJ, Brazil; danialviano@micro.ufrj.br (D.S.A.); alviano@micro.ufrj.br (C.S.A.)

**Keywords:** monohexosylceramides, *Rhizopus*, biofilm, antibacterial activities

## Abstract

Monohexosylceramides (CMHs) are highly conserved fungal glycosphingolipids playing a role in several cellular processes such as growth, differentiation and morphological transition. In this study, we report the isolation, purification and chemical characterization of CMHs from *Rhizopus stolonifer* and *R. microspores*. Using positive ion mode ESI-MS, two major ion species were observed at *m*/*z* 750 and *m*/*z* 766, respectively. Both ion species consisted of a glucose/galactose residue attached to a ceramide moiety containing 9-methyl-4,8-sphingadienine with an amidic linkage to a hydroxylated C16:0 fatty acid. The antimicrobial activity of CMH was evaluated against Gram positive and Gram negative bacteria using the agar diffusion assay. CMH from both *Rhizopus* species inhibited the growth of *Bacillus terrae*, *Micrococcus luteus* (*M. luteus*) and *Pseudomonas stutzeri* (*P. stutzeri*) with a MIC_50_ of 6.25, 6.25 and 3.13 mg/mL, respectively. The bactericidal effect was detected only for *M. luteus* and *P. stutzeri*, with MBC values of 25 and 6.25 mg/mL, respectively. Furthermore, the action of CMH on the biofilm produced by methicillin-resistant *Staphylococcus aureus* (MRSA) was analyzed using 12.5 and 25 mg/mL of CMH from *R. microsporus*. Total biofilm biomass, biofilm matrix and viability of the cells that form the biofilm structure were evaluated. CMH from *R. microsporus* was able to inhibit the MRSA biofilm formation in all parameters tested.

## 1. Introduction

Fungi of the order Mucorales are commonly found in soil as saprophytes and decomposing organic matter all around the world. In Brazil these fungi are widely found in the northeastern region in arid and semi-arid ecosystems such as the Caatinga, which is an exclusively Brazilian domain in the semi-arid region of Brazil [1,2]. Some species have been described as agents of systemic infections in humans, especially in immunocompromised patients [3,4]. *Rhizopus* species are frequently and amply distributed in soils of the Caatinga in the Brazilian Northeast [2]. Little was known about the cell wall glycoconjugates of the *Rhizopus* species and, consequently, their specific functions in the fungal cell and in their interaction with the environment.

Ceramide monohexosides (CMHs) are highly conserved fungal glycosphingolipids with structural modifications that include different sites of unsaturation as well as fatty acid residues of varying length in their ceramide moieties [5]. CMHs play diverse roles in fungal cell processes, such as growth and morphological transition in *Cryptococcus neoformans*, *Pseudallescheria boydii*, *Candida albicans*, *Aspergillus fumigatus* and *Collectotrichum gloeosporioides* [6,7,8,9,10]. In addition, CMH are important in promoting alkaline tolerance in vitro due to the involvement of CMH in the regulation of membrane fungal lipid domains that lead to the redistribution of CMH in the membrane in an alkaline environment [11,12,13]. CMH also interact with defensins isolated from insects and plants [14]. Studies on CMH with antimicrobial activity have been performed by isolating them from plants and microorganisms, such as fungi, where they play a role in the microbial growth [15,16,17,18].

Bacterial infectious diseases are highly prevalent in tropical and developing countries such as Brazil, affecting mainly hospitalized and/or immunosuppressed individuals [19,20,21]. *Staphylococcus aureus* is one of the most frequently found bacteria and is the etiological agent of nosocomial infections that is associated with the production of biofilm on medical implants. Furthermore, it possesses a strong capacity to acquire resistance resulting in high rates of therapeutic failure [22,23]. In addition, several environmental bacteria, such as *Pseudomonas stutzeri* and *Micrococcus luteus*, have emerged as opportunistic pathogens due to the increase in the number of immunosuppressed individuals, either by immunosuppressive treatment after transplantation, HIV infection or cancer [24,25,26,27].

In this context, the goal of this work was to characterize CMHs from two species of *Rhizopus*, *R. stolonifer* and *R. microsporus*, and to evaluate the ability of these highly conserved fungal glycosphingolipids to either inhibit the growth or to kill four different bacterial species. The influence of both *Rhizopus* CMHs in methicillin-resistant *S. aureus* (MRSA) biofilm formation was also studied.

## 2. Results

### 2.1. CMH Purification and Chemical Analysis

**Figure 1:**
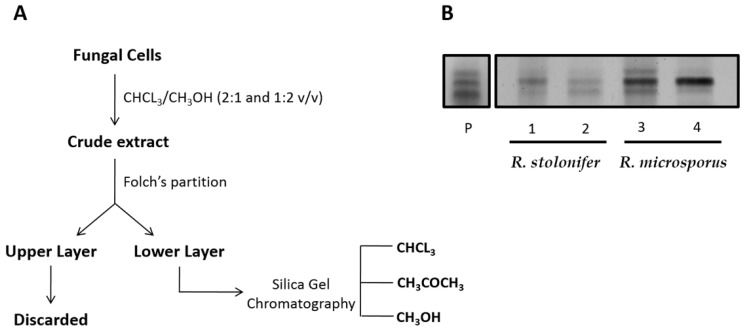
(**A**) Isolation and purification of glycosphingolipids from *Rhizopus* species. (**B**) High performance thin layer chromatography (HPTLC) of neutral glycosphingolipid fractions by silica gel column chromatography. P. CMH standard. Lane 1: Methanol fraction from *R. stolonifer* grown in potato dextrose broth (PDB). Lane 2: Methanol fraction from *R. stolonifer* grown in yeast extract-peptone-dextrose growth medium (YEPD). Lane 3: Methanol fraction from *R. microsporus* grown in PDB. Lane 4: Methanol fraction from *R. microspores* grown in YEPD. Solvent system: Chloroform/methanol/2 M NH_4_OH (40:10:1 *v*/*v*/*v*). Detection: iodine vapor and orcinol-sulfuric acid reagent.

The purification steps of CMH from the *Rhizopus* species grown in different media are shown in Figure 1A. The Folch lower layer containing neutral glycolipids was fractionated by silica gel chromatography and the fractions monitored by HPTLC on silica plates developed with chloroform: methanol: 2 M NH_4_OH (40:10:1 *v*/*v*). The spots were stained by spraying with orcinol-sulfuric acid reagent (Figure 1B).

**Figure 2:**
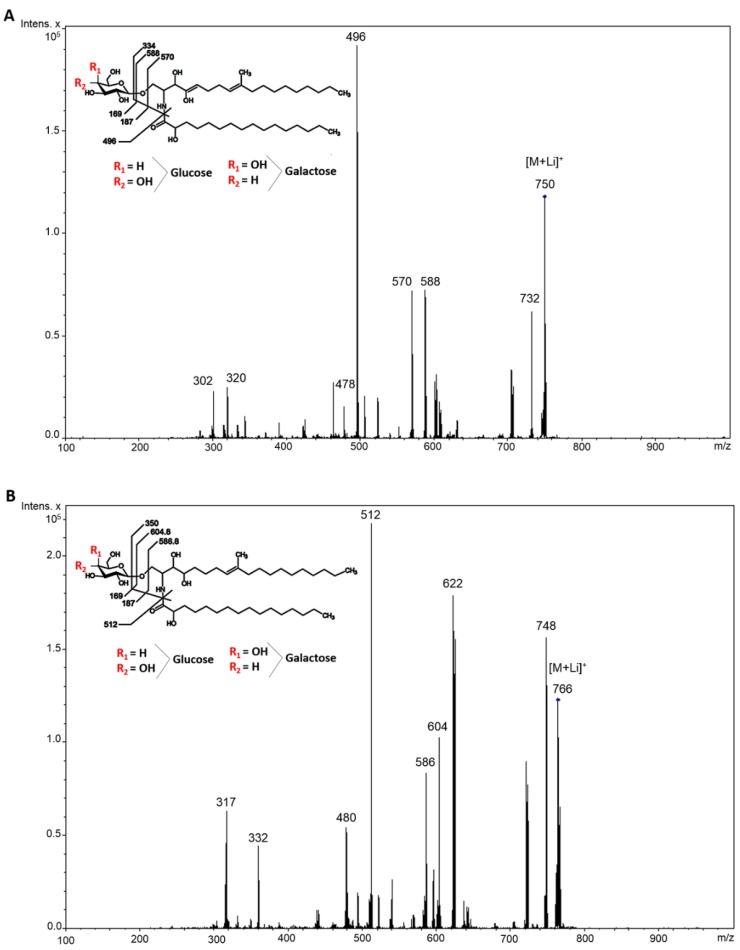
ESI-MS [M + Li] analysis of GlcCer/GalCer from *Rhizopus* species. (**A**) CMH from *R. microsporus* grown in PDB and YEPD, and CMH from *R. stolonifer* grown in YEPD. Major ions species at *m*/*z* 750. (**B**) CMH from *R. stolonifer* grown in PDB. Major ions species at *m*/*z* 766.

An ESI-MS analysis (positive mode) was performed to elucidate the chemical structure of purified CMHs from *Rhizopus* species. Lithiated molecular ions were observed at *m*/*z* 750 and *m*/*z* 766. A major lithiated ion species at *m*/*z* 750 was observed in *R. microsporus* grown in PDB and YEPD media and in *R. stolonifer* grown in YEPD medium, respectively. When submitted to the ESI-MS/MS analysis, these major peaks exhibited a fragment at *m*/*z* 732 (loss of water) and daughter ions at *m*/*z* 588 and *m*/*z* 570 (loss of water). The loss of 162 mass units common to the CMHs analyzed corresponds to the loss of a hexose residue (glucose or galactose) (Figure 2A). The prominent ion at *m*/*z* 496 suggests the presence of an extra hydroxyl group in the long chain base [9]. CMH from *R. stolonifer* grown in PDB medium presents a major lithiated species at *m*/*z* 766. The loss of 162 units generated daughter ions at *m*/*z* 622 [M-hexose + Li^+^] and *m*/*z* 604 [M-hexose-H_2_O^+^ Li^+^] corresponding to the ceramide monolithiated ion (Figure 2B). According to these results, we concluded that the glycosphingolipid structures consisted of a hexose, long-chain bases (4-OH-9-methyl-octadeca-4,8-sphingadienine and 4-OH-9-methyl-eicosa-4,8-sphingadienine) and hydroxylated C16:0 fatty acids. After hydrolysis with 3 M TFA, the constituent monosaccharides were identified by GC to be glucose and galactose in a ratio 1:1 from both *Rhizopus* species studied.

### 2.2. Antibacterial Activity Evaluation

The antibacterial activity of CMHs from *Rhizopus* species was evaluated against six different species of indicator bacteria using the agar diffusion assay (Table 1 and Figure S1). In this assay, the lower layer from Folch partition and CMH from both species (50 mg/mL–10 µL), were able to inhibit the growth of *B. terrae*, *M. luteus* and *P. stutzeri*, but there was no inhibition of other bacteria tested (*B. cepacea*, *E. coli*, *S. aureus* and MRSA-*S. aureus*). The lower layer from Folch partition containing the neutral lipids from the crude lipid extract shows lower inhibition when compared with the purified CMH.

**Figure 3:**
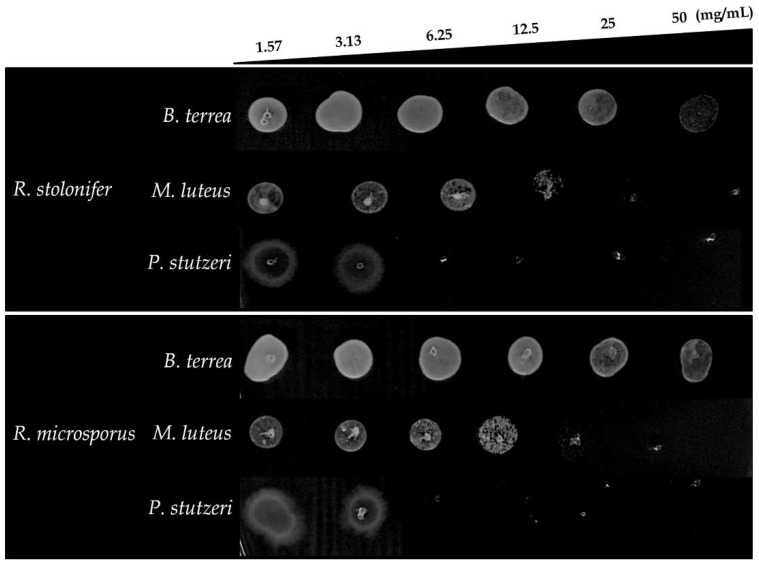
Minimal Bactericidal Concentration (MBC) of CMHs from *Rhizopus* species against *B. terrea*, *M. luteus* and *P. stutzeri* analyzed by dot spot technique.

To confirm the results obtained in the agar diffusion assay, the Minimum Inhibitory Concentration (MIC) and Minimum Bactericidal Concentration (MBC) values of CMH were determined against *B. terrae*, *M. luteus* and *P. stutzeri* (Table 2). CMHs from both species showed considerable inhibitory activity, with MIC_50_ of 6.25 and 3.13 mg/mL for *M. luteus* and *P. stutzeri*, respectively. CMH from *R. stolonifer* with MIC_50_ values of 6.25 mg/mL against *B. terrae* and 3.13 mg/mL for CMH from *R. microsporus*. *P. stutzeri* was most sensitive to CMH, resulting in the lowest MIC_50_ and MBC values of 6.25 mg/mL, followed by *M. luteus* with MIC_50_ of 6.25 and MBC of 25 mg/mL. However, CMHs did not show antimicrobial activity against *B. terrae* (MBC > 50 mg/mL) (Figure 3). Metabolic activity of the bacteria was measured using XTT-assay for all concentration tested (1.57 to 50 mg/mL). CMHs from *R. stolonifer* and *R. microsporus* were able to reduce the viability more than 50% at the concentration of 6.25 mg/mL. Streptomycin/Penicillin were used as standard references for activity against *B. terrae*, *M. luteus* and *P. stutzeri*.

### 2.3. Evaluation of Anti-Biofilm Activity

**Figure 4:**
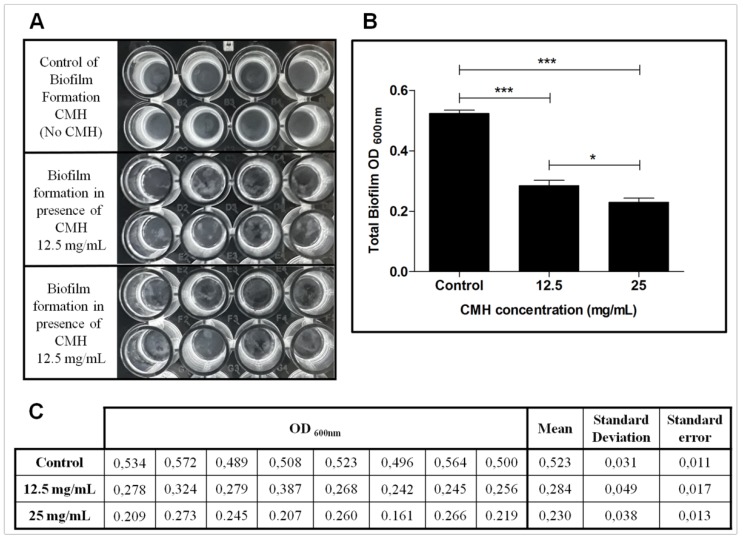
Effect of CMH on MRSA-*S. aureus* biofilm formation. (**A**) Representative biofilm formation at different concentration of CMH (12.5 and 25 mg/mL) and control. (**B**) Quantification of total biofilm formed in presence or absence of CMH. Absorbance determined at 600 nm. (**C**) CMH effect on MRSA biofilm formation determined by absorbance measurement at 600 nm. Mean, standard deviation and standard error are represented. Statistical differences (* *p* < 0.05, ** *p* < 0.001, *** *p* < 0.0001) are represented by asterisks (ns = not significant).

**Figure 5:**
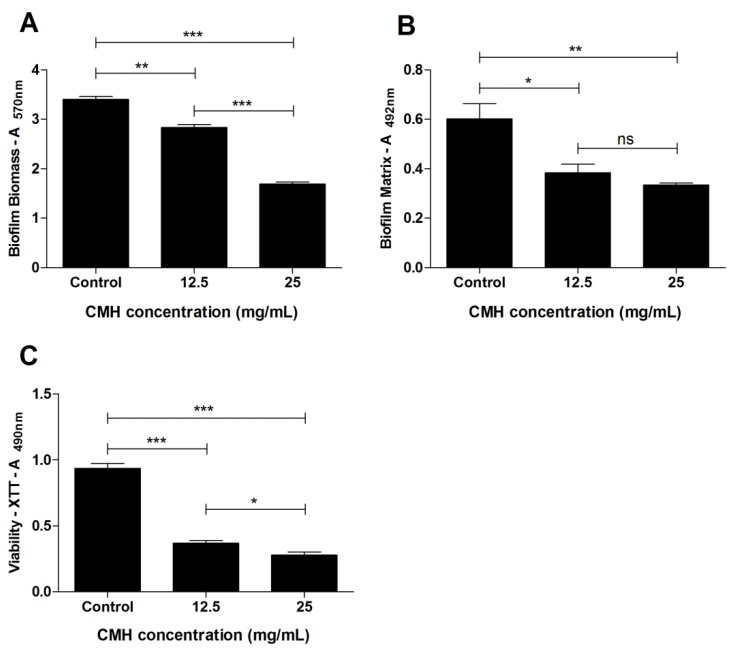
Effect of CMH on MRSA-*S. aureus* biofilm formation, after 24 h of growth. (**A**) Total biomass of the biofilm was quantified by crystal violet assay. (**B**) The biofilm matrix was quantified by safranin assay. (**C**) Metabolic activity of the cells inside the biofilm was detected by XTT-reduction assay. Statistical differences (* *p* < 0.05, ** *p* < 0.001, *** *p* < 0.0001) are represented by asterisks (ns = not significant) ((**A**) = absorbance). Control represent MRSA growth in absence of CMH. Values represent the mean ± S.D. of three independent experiments performed in triplicate.

The effect of CMH (*m*/*z* 750) from *R. microsporus* on the biofilm produced by methicillin resistant *S. aureus* (MRSA) was analyzed using two different concentrations (12.5 and 25 mg/mL). CMH was not able to inhibit the growth of MRSA, however it could interfere with biofilm formation. Therefore, *S. aureus* (MRSA) was chosen as indicator bacterium due to its multi-drug resistance and ability to form biofilm. Representative biofilms formed by MRSA-*S. aureus* are shown in Figure 4A and the absorbance was determined at 600 nm (Figure 4B). Absorbance values, mean, standard deviation and standard error are represented in Figure 4C. Biofilm production on polystyrene microplates was also analyzed by correlating the results of three different analyses: The total biofilm biomass (including biofilm structure depleted of planktonic cells) was evaluated using crystal violet assay (Figure 5A, Table S1). The production of extracellular matrix was, evaluated by safranin assay (Figure 5B, Table S1), and the metabolic activity of the biofilm was quantified using XTT-assay (Figure 5C, Table S1). CMH from *R. microsporus* was able to reduce the total biofilm biomass in both concentrations tested. The biofilm matrix was also reduced and the metabolic activity of the biofilm was drastically decreased. Although CMH was not able to kill MRSA, it was could capably interfere on the biofilm structure formation.

## 3. Discussion

Glycosphingolipids are membrane lipids distributed among all eukaryotic cells. They are highly enriched in the plasma membrane and are present in membrane microdomains together with sterols and proteins. Glycosylceramides are the main neutral glycosphingolipids expressed in fungal pathogens [7,28,29], and may have glucose and galactose as glycan motifs [30]. Glycosphingolipids play an important role in relevant cellular functions such as cell growth, cell adhesion and motility, carbohydrate-carbohydrate interactions and also act as potential antifungal agents [31,32]. In this work, CMHs obtained from *R. stolonifer* and *R. microsporus* grown in PDB or YEPD medium had their chemical structures elucidated by electrospray ionization mass spectrometry (ESI-MS) and by gas chromatography (GC). In this work, the role of CMH in inhibiting the growth of several bacterial species or the biofilm formation in methicillin-resistant *S. aureus* (MRSA) was also studied.

The major glycosphingolipids detected in *Rhizopus* species grown in PDB and YEPD are *N*-2′-hydroxyhexadecanoyl-1-*O*-β-d-glucopyranosyl-4-OH-9-methyl-octadeca-4,8-sphingadienine and *N*-2′-hydroxyhexadecanoyl-1-*O*-β-d-galactopyranosyl-4-OH-9-methyl-octadeca-4,8-sphingadienine with a ratio of 1:1. The main difference between *R. stolonifer* CMHs grown in PDB medium and the other *Rhizopus* species described above is the presence of 4-OH-9-methyl-eicosa-4,8-sphingadienine as the main long chain base. To our knowledge, this is the first time that different CMHs structures were identified in *R*. *stolonifer* grown in different culture media. CMHs with ceramide mono-, di-, tri-, tetra- and pentasaccharides have been found in other *Zygomycetes* species as *Mucor hiemalis* [33].

Currently, many clinically important and emerging pathogens are resistant to all or almost all antibiotics tested. This is a serious public health problem with great medical and social dimensions. The indiscriminate use of antibiotics has greatly increased the number of different bacterial species resistant to antimicrobials, which are normally used in clinical settings [34]. Therefore, there is an urgency for studies looking for the discovery of new antimicrobial agents.

In this context, CMHs isolated from *R. stolonifer* and *R. microsporus* grown in PDB were tested against both pathogenic and opportunistic bacteria, such as *B. cepacea*, *B. terrae* and *E. coli* (Gram negative bacteria), and *M. luteus*, *P. stutzeri* and *S. aureus* (Gram positive bacteria). In the agar diffusion assay, 50 mg/mL of the Folch lower phase (containing neutral lipids) or purified CMH from *Rhizopus* species showed antimicrobial activity against *B. terrae*, *M. luteus* and *P. stutzeri*. The growth of *B. cepacea*, *E. coli*, *S. aureus* and MRSA was not affected by either the Folch lower phase or by CMH. The best antimicrobial potential was observed for CMH from both fungi, with MIC_50_ values of 3.13 mg/mL against *P. stutzeri*, followed by *M. luteus* with MIC_50_ 6.25 mg/mL. The MIC values for CMH from *R. stolonifer* against *B. terrae* was 6.25 mg/mL and from *R. microsporus* was 3.13 mg/mL. The CMHs from both fungi were able to decrease the viability of the three indicator bacteria tested. However, the bactericidal activity of CMH was only detected against *M. luteus* and *P. stutzeri*, with MBC values of 25 and 6.25 mg/mL, respectively. Previous work has shown that glycosphingolipids, which are amphipathic molecules, may present several biological activities, such as antimicrobial and biosurfactant actions [35].

An important survival strategy of microorganisms is the formation of biofilm, which is considered an adaptation to hostile environmental conditions [36]. Biofilms can be defined as a community of cells attached to a substrate embedded in a matrix of extracellular polymeric substances. Inside the structure of the biofilm, channels are formed that allow the passage of water, oxygen and nutrients [37]. The biofilm produced by *S. aureus* is an important virulence factor associated with many localized infections. In this work, although CMH did not inhibit the growth of MRSA, we investigated whether it would be able to inhibit the formation or disaggregation of the biofilm structure formed by MRSA. The biofilm produced by MRSA in the presence or absence of CMH was evaluated by three parameters: Total biofilm biomass, biofilm matrix and viability of the biofilm-forming cells. The results showed that both CMH concentrations (12.5 and 25 mg/mL) were able to influence all tested parameters. Biosurfactants isolated from *Lactobacillus jensenii* and *L. rhamnosus* showed anti-biofilm activities against clinical Multidrug Resistant (MDR) strains of *Acinetobacter baumannii*, *E. coli*, and *S. aureus* (MRSA) at the concentrations of 25 and 50 mg/mL [38]. However, the chemical structure of these molecules has not been elucidated.

CMH could inhibit MRSA adhesion which represents the initial stage of biofilm formation [35]. However, studies are needed to investigate possible synergism between CMHs from *Rhizopus* species and the antibiotics used routinely for the treatment of MRSA infections.

## 4. Material and Methods

### 4.1. Microorganisms and Culture Conditions

*Rhizopus stolonifer* UPC1300 and *Rhizopus microsporus* var. *microsporus* UPC1304, isolated from the Brazilian Caatinga area, were supplied by the Culture Collection (RENNORFUN) from the Catholic University of Pernambuco, Recife, Brazil. Strains were maintained in Sabouraud agar solid medium under refrigeration at 4 °C. Cells were inoculated in Erlenmeyer flasks containing 200 mL of potato dextrose broth (PDB) and/or YEPD (glucose 2%, peptone 2%, yeast extract 1%), and incubated at room temperature for 7 days with orbital shaking (pre-inoculum). Cultures (200 mL) were then transferred to the same medium (3 L) and incubated for a further 7 days at the same temperature with shaking. At the end, the mycelium was filtered, washed with distilled water, and stored at −20 °C.

### 4.2. Extraction and Purification of CMH from R. stolonifer and R. microsporus

Total lipids from intact hyphae of *R. stolonifer* and *R. microsporus* grown in PDB or YEPD were successively extracted at room temperature using chloroform/methanol at ratios 2:1 and 1:2 (*v*/*v*), as described [7]. The crude lipid extracts were partitioned with chloroform/methanol/ 0.45% KCl (8:4:3 *v*/*v*) as described by Folch et al. (1957) [39]. The Folch lower layer containing neutral lipids, glycosphingolipids and phospholipids was fractionated on a silica gel column which was sequentially eluted with chloroform, acetone and then methanol [12]. The recovery of glycosphingolipids by elution with methanol was monitored by thin-layer chromatography (TLC), on silica gel plates developed with chloroform/methanol/2 M ammonium hydroxide, 40:10:1 (*v*/*v*). The spots were visualized with iodine and by charring with orcinol/H_2_SO_4_.

### 4.3. Sugar Analysis

Glycosphingolipids were hydrolyzed with 3 M trifluoroacetic acid at 100 °C for 3 h and the released monosaccharides were characterized by HPTLC developed with n-butanol/acetone/water (4:5:1 *v*/*v*) and visualized by spraying with orcinol-sulfuric acid reagent. The monosaccharides were converted to their alditol acetate derivatives and quantified by GC using the chromatograph GC-2010 Plus-Shimadzu, with a SH-RTX-5 capillary column (30 m, 0.25 mm ID, 0.25 Um df), programmed for an initial isothermal period of 10 min at 190 °C, and subsequent temperature increase of 2 °C/min, until 210 °C were reached [40,41].

### 4.4. ESI-MS Analysis

CMHs were analyzed by electrospray ionization (ESI-MS) in positive (ESI+) ion mode, using an ESI-ion Trap instrument (Model Amazon SL, Bruker, Germany). CMHs were diluted in chloroform/methanol/water (5:4:1 *v*/*v*), containing 1 mM lithium chloride and analyzed via direct injection using a microsyringe pump (Hamilton) [42]. Nitrogen was used as nebulizer and carrier gas.

### 4.5. Antimicrobial Assay

#### 4.5.1. Bacterial Strains

*Burkholderia cepacea* (American Type Culture Collection, ATCC 25416), *B. terrea* BS001 (Microbial Ecology culture collection, University of Groningen, Groningen, The Netherlands), *Escherichia coli* (American Type Culture Collection, ATCC11229), *Micrococcus luteus* (American Type Culture Collection, ATCC4698) Methicillin Resistant *Staphylococcus aureus*-MRSA (American Type Culture Collection, ATCC9393), *Pseudomonas stutzeri* R55 (Laboratory of Molecular Microbial Ecology and Microbial Diversity Culture Collection, Instituto de Microbiologia Paulo de Góes, Universidade Federal do Rio de Janeiro/UFRJ). Bacteria were grown at 37 °C in Luria-Bertani (LB) broth (peptone 10 g/L, yeast extract 5 g/L and NaCl 5 g/L).

#### 4.5.2. Antimicrobial Activity Assay

Antimicrobial activity of CMH [50 mg/mL in chloroform/methanol (2:1 *v*/*v*)] was determined against all bacterial strains by the agar diffusion method with modifications. Briefly, 10 µL of CMH (50 mg/mL) were spotted on a Petri dish containing LB agar medium. After evaporation of the organic solvents, an inoculum of each bacterial strain containing approximately 1.5 × 10^8^ colony forming units (CFU/mL) prepared in Luria-Bertani broth medium was spread out on the plates, and the plates were incubated for 24 h at 37 °C. A positive control with inoculum without CMH was included. Growth inhibition zones were evidenced using MTT (Thiazolyl Blue Tetrazolium Bromide—Sigma Aldrich, St. Louis, MO, USA) [43].

#### 4.5.3. Determination of MIC and MBC

MIC was evaluated by the dilution method in LB broth. CMH concentrations ranging from 15.7 mg/mL to 50 mg/mL were tested and 10 μL of the bacterial suspension containing 1.5 × 10^8^ CFU/mL (0.1 OD_600 nm_) were added to each well and incubated at 37 °C for 24 h. DMSO was used as control (5% in LB broth medium) and streptomycin/penicilin was used as a reference compound (8–0.015 µg/mL). After 24 h, the plates were read at 600 nm to evaluate the MIC_50_. After MICs reading, MBC (minimal bactericidal concentration) was determined by subculturing an aliquot of 10 µL from each well that showed complete growth inhibition in LB agar medium without addition of CMH and the bacterial growth was evaluated for the MBC determination. After 24 h, the MBC values were defined as the lowest concentration of CMH able of eliminate bacteria [44]. The experiments were carried out in duplicates. Following the MIC assays, cell viability was evaluated using XTT reduction assay (XTT sodium salt—0.5 mg/mL—Sigma Aldrich, St. Louis, MO, USA) [45].

#### 4.5.4. Effect of CMH on Inhibition of MRSA ATCC9393 Biofilm

50 μL of MRSA ATCC9393 suspension (0.1 OD_600 nm_) in Tryptic Soy Broth (TSB) supplemented with 1% glucose were mixed with 50 µL of CMH (12.5 and 25 mg/mL) from *R. microspores* and added to each well of a 96-well polystyrene plate. Culture medium with the bacterial suspension was used as negative control. After 24 h incubation at 37 °C, the formed biofilm was gently washed with sterile distilled water to remove planktonic cells, air-dried for 10 min and stained with 0.5% crystal violet for 10 min (total biofilm biomass) or 1% safranin for 10 min (biofilm matrix). The staining solutions were discarded and the biofilms were rinsed gently twice with sterile distilled water. The crystal violet impregnated in the biofilm was dissolved in 200 µL of ethanol (95%, *v*/*v*), and the colored solution was read at an absorbance of 595 nm using a spectrophotometer (Spectra MAX 340 Tunable; Molecular Devices Ltd., San Jose, CA, USA). Safranin was dissolved in water (100 µL), and the absorbance read at 492 nm. Viability analysis of the cells in the biofilm was performed by XTT reduction assay. Biofilms were washed with sterile distilled water, and 150 µL of XTT solution were added to each well. The XTT solution was prepared by dissolving 5 mg of XTT in 10 mL of water, followed by the addition of 400 μL Menadione solution (0.17 mg/mL in acetone). After incubation at 37 °C for 1 h under protection from light, the absorbance of the colored solution was measured at 490 nm.

### 4.6. Statistical Analysis

All statistical analyses were performed using GraphPad Prism 5.0 software (GraphPad, San Diego, CA, USA). A variance two-way ANOVA was performed using Tukey’s and Bonferroni’s comparisons tests to evaluate the biofilm formation.

## Figures and Tables

**Table 1:** Antimicrobial Activity of CMHs from *Rhizopus* species by Agar Diffusion Assay. Lipid samples (Folch lower layer and CMH) from *R. stolonifer* and *R. microsporus* grown in PDB were dissolved in chloroform/methanol (2:1 *v*/*v*) at a concentration of 50 mg/mL (10 µL). The negative control consisted of 10 µL of chloroform/methanol (2:1 *v*/*v*).

*Rhizopus* Species	Bacteria Species	Inhibition (+) or No Inhibition (−)		
		Control	Folch Lower Layer	CMH
*R. stolonifer*	*B. cepacea*	−	−	−
	*B. terrea*	−	±	+
	*E. coli*	−	−	−
	*M. luteus*	−	±	+
	*S. aureus*	−	−	−
	*S. aureus* MRSA	−	−	−
	*P. stutzeri*	−	+	+
*R. microsporus*	*B. cepacea*	−	−	−
	*B. terrea*	−	+	±
	*E. coli*	−	−	−
	*M. luteus*	−	±	±
	*S. aureus*	−	−	−
	*S. aureus* MRSA	−	−	−
	*P. stutzeri*	−	+	+

**Table 2:** Minimum Inhibitory Concentration (MIC) and Minimum Bactericidal Concentration (MBC) values from CMH of *R. stolonifer* and *R. microsporus* grown in PDB media. MIC values from Streptomycin/Penicillin as a drug control.

CMH Fraction	Bacteria	MIC_50_	MBC
*R. stolonifer*	*B. terrea*	6.25 mg/mL	>50 mg/mL
	*M. luteus*	6.25 mg/mL	25 mg/mL
	*P. stutzeri*	3.13 mg/mL	6.25 mg/mL
*R. microsporus*	*B. terrea*	3.13 mg/mL	>50 mg/mL
	*M. luteus*	6.25 mg/mL	25 mg/mL
	*P. stutzeri*	3.12 mg/mL	6.25 mg/mL
Streptomycin/Penicillin	*B. terrea*	0.004 mg/mL	-
	*M. luteus*	0.004 mg/mL	-
	*P. stutzeri*	0.004 mg/mL	-

MIC_50_ Minimal Inhibitory Concentration; MBC Minimal Bactericidal Concentration.

## Supplementary Materials

The following are available, **Figure S1:** Antimicrobial Activity of CMHs from *R. stolonifer* and *R. microsporus* against a panel of Gram-positive and Gram-negative bacterium by agar disc diffusion method. CMH or Folch lower layer (Folch↓) were dissolved in chloroform/methanol (2:1 *v*/*v*) at a concentration of 50 mg/mL. Each spot contained 10 μL. The control spot contained 10 µL of chloroform/methanol (2:1 *v*/*v*). Growth inhibition zones were evidenced by MTT assay. **Table S1:** Effect of CMH on MRSA-*S. aureus* biofilm formation. Analyses were done correlating the results of total biofilm biomass (evaluated by crystal violet assay, A_570 nm_), production of extracellular matrix (evaluated by safranin assay, A_492 nm_) and metabolic activity (quantified by XTT-assay, A_490 nm_). Mean, standard deviation (SD) and standard error (SE) are also shown. (A = absorbance).
