# Supplementary material for: Monohexosylceramides from Rhizopus Species Isolated from Brazilian Caatinga: Chemical Characterization and Evaluation of Their Anti-Biofilm and Antibacterial Activities
Source: Molecules. 2018 Jun 1;23(6):1331. doi: 10.3390/molecules23061331 (PMC6100016; doi:10.3390/molecules23061331)
Supplement: Supplementary file 1 [file molecules-23-01331-s001.zip › Supplementary Table 1.docx]

| Biofilm biomass  Crystal Violet A _570nm_ | | | Extracellular matrix  Safranin A _492nm_ | | | Cell viability – XTT  A _490nm_ | | |
| --- | --- | --- | --- | --- | --- | --- | --- | --- |
| Control | 12.5 mg/ml | 25 mg/ml | Control | 12.5 mg/ml | 25 mg/ml | Control | 12.5 mg/ml | 25 mg/ml |
| 3.485 | 3.163 | 1.745 | 0.509 | 0.417 | 0.301 | 1.080 | 0.342 | 0.221 |
| 3.286 | 3.147 | 1.587 | 0.659 | 0.454 | 0.334 | 0.953 | 0.493 | 0.296 |
| 3.357 | 2.811 | 1.728 | 0.623 | 0.308 | 0.349 | 0.914 | 0.371 | 0.389 |
| 3.208 | 2.600 | 1.489 | 0.559 | 0.317 | 0.331 | 0.960 | 0.351 | 0.258 |
| 3.210 | 2.690 | 1.534 | 0.851 | 0.499 | 0.334 | 0.782 | 0.339 | 0.229 |
| 3.276 | 2.929 | 1.758 | 0.409 | 0.308 | 0.357 | 1.005 | 0.352 | 0.222 |
| 3.499 | 2.763 | 1.555 | ̶ | ̶ | ̶ | 0.856 | 0.337 | 0.337 |
| 3.230 | 2.722 | 1.853 | ̶ | ̶ | ̶ | ̶ | ̶ | ̶ |
| 3.629 | 2.839 | 1.783 | ̶ | ̶ | ̶ | ̶ | ̶ | ̶ |
| 3.830 | 2.736 | 1.877 | ̶ | ̶ | ̶ | ̶ | ̶ | ̶ |
| Mean 3,401 | Mean  2,840 | Mean  1,691 | Mean  0,6017 | Mean  0,3838 | Mean  0,3343 | Mean  0,9357 | Mean  0,3693 | Mean  0,2789 |
| SD  0,2072 | SD  0,1880 | SD  0,1386 | SD  0,1506 | SD  0,08397 | SD  0,01924 | SD  0,09743 | SD  0,05575 | SD  0,06494 |
| SE  0,06553 | SE  0,05945 | SE  0,04382 | SE  0,06149 | SE  0,03428 | SE  0,007856 | SE  0,03683 | SE  0,02107 | SE  0,02455 |

**Supplementary Table 1.** Effect of CMH on MRSA - *S. aureus* biofilm formation. Analyses were done correlating the results of total biofilm biomass (evaluated by crystal violet assay, A _570nm_), production of extracellular matrix (evaluated by safranin assay, A _492nm_) and metabolic activity (quantified by XTT-assay, A _490nm_). Mean, standard deviation (SD) and standard error (SE) are also shown. (A = absorbance).
