# Supplementary figures and images for: Monohexosylceramides from Rhizopus Species Isolated from Brazilian Caatinga: Chemical Characterization and Evaluation of Their Anti-Biofilm and Antibacterial Activities
Source: Molecules. 2018 Jun 1;23(6):1331. doi: 10.3390/molecules23061331 (PMC6100016; doi:10.3390/molecules23061331)

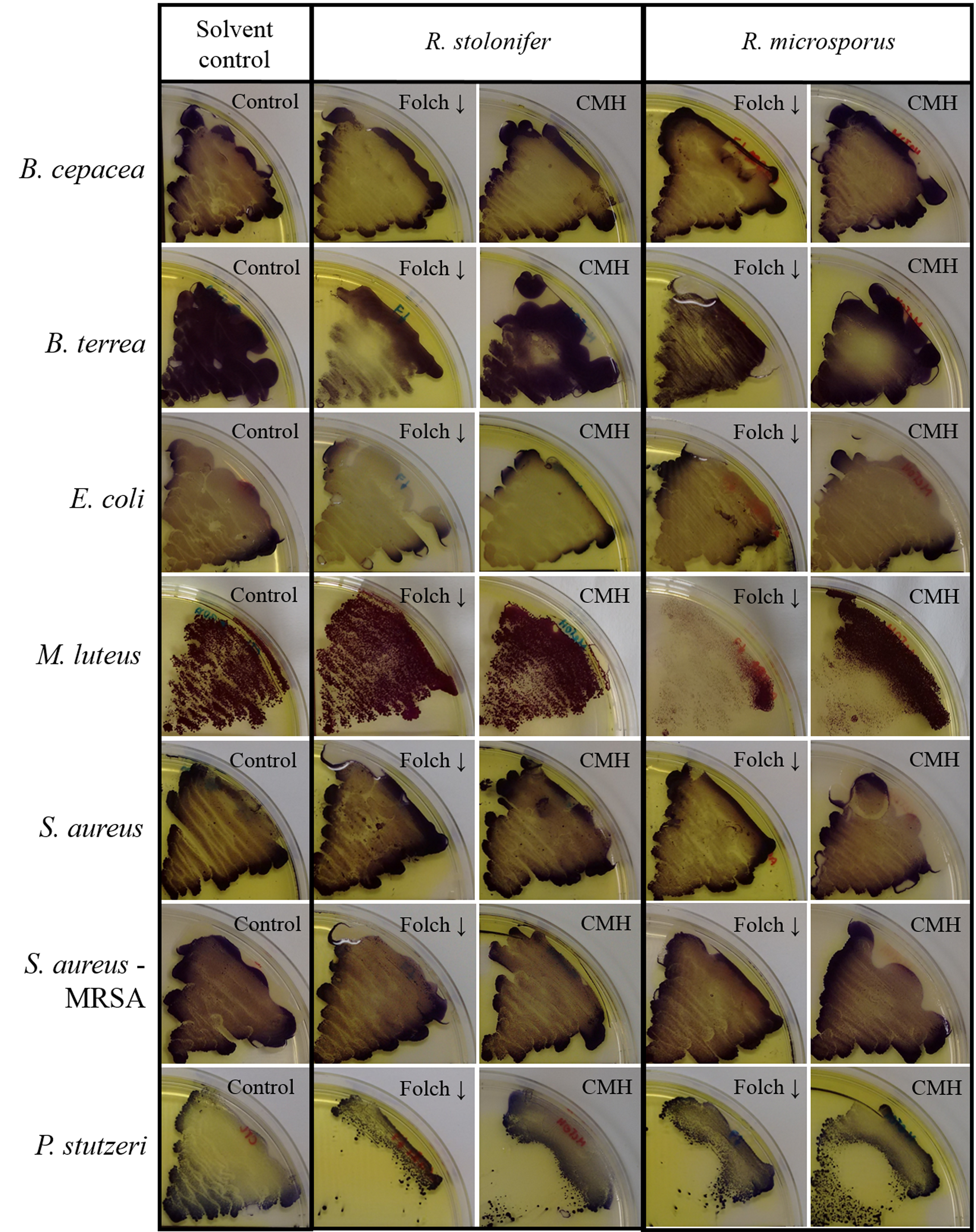

Supplement: Supplementary file 1 [file molecules-23-01331-s001.zip › Supplementery figure 1.png]
